# Effects of neuroactive metabolites of the tryptophan pathway on working memory and cortical thickness in schizophrenia

**DOI:** 10.1038/s41398-021-01311-z

**Published:** 2021-04-01

**Authors:** Junchao Huang, Jinghui Tong, Ping Zhang, Yanfang Zhou, Yimin Cui, Shuping Tan, Zhiren Wang, Fude Yang, Peter Kochunov, Joshua Chiappelli, Baopeng Tian, Li Tian, Yunlong Tan, L. Elliot Hong

**Affiliations:** 1 Peking University HuiLongGuan Clinical Medical School, Beijing Huilongguan Hospital, Beijing, China; 2 Department of Pharmacy, Peking University First Hospital, Beijing, China; 3 Maryland Psychiatric Research Center, Department of Psychiatry, University of Maryland School of Medicine, Baltimore, MD USA; 4 Faculty of Medicine, Department of Physiology, Institute of Biomedicine and Translational Medicine, University of Tartu, Tartu, Estonia

**Keywords:** Schizophrenia, Learning and memory

## Abstract

A number of tryptophan metabolites known to be neuroactive have been examined for their potential associations with cognitive deficits in schizophrenia. Among these metabolites, kynurenic acid (KYNA), 5-hydroxyindole (5-HI), and quinolinic acid (QUIN) are documented in their diverse effects on α-7 nicotinic acetylcholine receptor (α7nAChR) and/or *N*-methyl-D-aspartate receptor (NMDAR), two of the receptor types thought to contribute to cognitive impairment in schizophrenia. In this study, serum levels of KYNA, 5-HI, and QUIN were measured in 195 patients with schizophrenia and in 70 healthy controls using liquid chromatography-tandem mass spectrometry; cognitive performance in MATRICS Consensus Cognitive Battery and cortical thickness measured by magnetic resonance imaging were obtained. Patients with schizophrenia had significantly lower serum KYNA (*p* < 0.001) and QUIN (*p* = 0.02) levels, and increased 5-HI/KYNA (*p* < 0.001) and QUIN/KYNA ratios (*p* < 0.001) compared with healthy controls. Multiple linear regression showed that working memory was positively correlated with serum 5-HI levels (*t* = 2.10, *p* = 0.04), but inversely correlated with KYNA concentrations (*t* = −2.01, *p* = 0.05) in patients. Patients with high 5-HI and low KYNA had better working memory than other subgroups (*p* = 0.01). Higher 5-HI levels were associated with thicker left lateral orbitofrontal cortex (*t* = 3.71, *p* = 2.94 × 10^−4^) in patients. The different effects of 5-HI and KYNA on working memory may appear consistent with their opposite receptor level mechanisms. Our findings appear to provide a new insight into the dynamic roles of tryptophan pathway metabolites on cognition, which may benefit novel therapeutic development that targets cognitive impairment in schizophrenia.

## Introduction

The tryptophan pathway has been increasingly targeted in drug discovery efforts for treating cognitive impairments in neuropsychiatric conditions including schizophrenia^1,2^. One of the metabolites in this pathway is kynurenic acid (KYNA), which has attracted considerable interest, as it is a non-competitive antagonist of α-7 nicotinic acetylcholine receptor (α7nAChR)^3,4^ that has been linked to cognitive functions, in particular working memory and attention^5,6^. Accumulated evidences suggest that schizophrenia is associated with abnormal α7nAChR-mediated neurotransmission^7,8^ and cortical KYNA contributes to cognitive impairment through the α7nAChR mechanism^3,9–11^. KYNA also antagonizes the glycine-binding site of the *N*-methyl-D-aspartate receptor (NMDAR)^4,12^ and NMDAR dysfunction has been linked to cognition, especially working memory deficits in schizophrenia^13–16^.

**Fig. 1:**
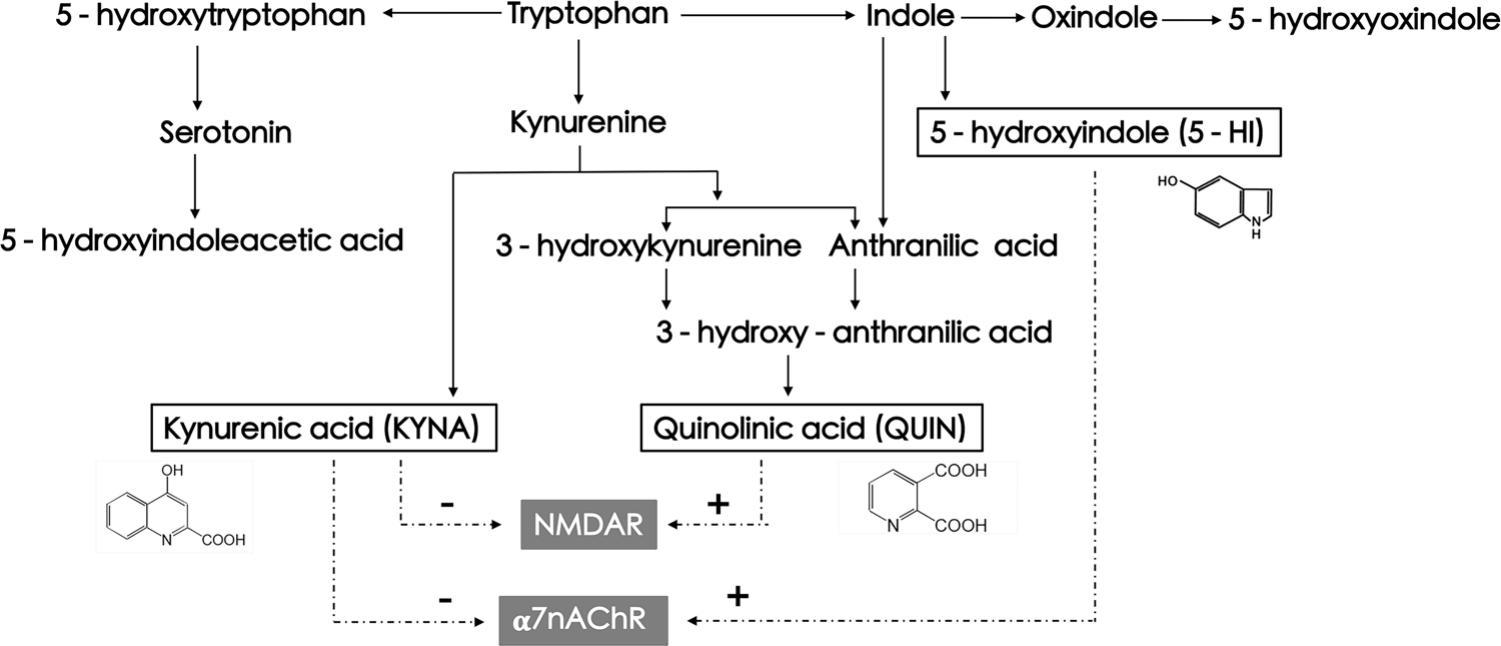
Abbreviated tryptophan pathway and the presumed neuroactive metabolite effects on α7nAChR and NMDAR. Dotted line refers to hypothetical effects based on preclinical data. Positive signs show the agonist effect and negative signs show the antagonist effect^17,49,89,90^.

Interestingly, another neuroactive metabolite in the tryptophan pathway, 5-hydroxyindole (5-HI), also affects α7nAChR^17–19^. 5-HI is a lipophilic solute and was shown to permeate epithelial plasma membrane^20^, implying that peripheral 5-HI may penetrate the blood–brain barrier (BBB). In contrast to KYNA, 5-HI potentiates α7nAChR-mediated electrophysiological responses and Ca^2+^ influx in a concentration-dependent manner^18,19,21^. Furthermore, 5-HI-potentiated α7nAChR activations are dose-dependently downregulated by KYNA^19^. Hence, these two metabolites of the tryptophan pathway counteract on α7nAChR-mediated functions and may form an opposing effect on working memory and other cognitive functions mediated by α7nAChR (Fig. 1).

5-HI and KYNA may also diverge on their roles in cognition, as they may modulate γ-aminobutyric acid (GABA)ergic neurotransmission differently, as 5-HI facilitates GABAergic transmission via excitation of α7nAChR^22^, but KYNA concentration-dependently reduces GABA levels by modulating α7nAChR function^23^. GABAergic dysfunction has also been consistently linked to working memory deficits in schizophrenia^24–26^.

Notably, a third metabolite in the tryptophan pathway, quinolinic acid (QUIN), is an agonist of NMDAR^27,28^. QUIN is an excitotoxin that promotes neurodegeneration and neuroinflammation, and may negatively impact cognition^29–31^.

Therefore, 5-HI, KYNA, and QUIN are three neuroactive metabolites within the tryptophan pathway, which may potentially influence cognition, although their in vivo combined effects are likely complex and are currently unknown. The effort of targeting the tryptophan pathway for treating cognitive deficits in schizophrenia should first understand the independent vs. joint effects of these neuroactive metabolites. Accordingly, we tested the hypothesis that working memory in schizophrenia may be dynamically dependent on the balance among 5-HI, KYNA, and QUIN. As these metabolites are known to influence α7nAChR and NMDAR^32,33^, which are distributed in high densities in the cortex^34,35^, we will test a second hypothesis that they may also counteractively impact the cortical thickness in patients with schizophrenia.

## Methods

### Participants

The study recruited 195 patients with schizophrenia (all in-patients) and 70 healthy controls (HCs). Patients who met the diagnostic criteria of schizophrenia according to the Structured Clinical Interview of the Diagnostic and Statistical Manual of Mental Disorders-IV were enrolled during their hospitalization at the Beijing Huilongguan Hospital. HCs were recruited through local advertisements at nearby communities and were excluded if they had a history of psychiatric disorders or psychosis among their first-degree relatives. Participants were also excluded if they had a lifetime history of any other Axis I disorders, head trauma, current or previous substance or alcoholism dependence (except nicotine), or systemic diseases including neurological disorders, organic brain disorders, and unstable medical illnesses. One hundred and twelve patients were antipsychotic medication-free at the time of admission and blood sample collection; 19 patients were on a first-generation antipsychotic alone or combined with second-generation antipsychotics; and the remaining patients were on the following second-generation antipsychotics: risperidone (14), clozapine (5), olanzapine (5), and aripiprazole (4). Thirty-six patients were on two or more second-generation antipsychotics and four patients were also on sodium valproate. Patients received antipsychotic medications once hospitalized and, most initially, medication-free patients were on antipsychotic medications during brain imaging, which was on average 5.5 ± 2.3 days after the initial blood draw. The current antipsychotic medication dose (based on time of blood draw) was calculated as a chlorpromazine (CPZ)-equivalent dose^36,37^ (Table 1). This study was approved by the ethics committee and institutional review board of Beijing Huilongguan Hospital. All participants provided written informed consent.

**Table 1:** Participant demographics, clinical characteristics, and metabolic measures.

	Schizophrenia (*n* = 195)	Healthy controls (*n* = 70)	Test statistic	*p*-Value
Male/female	110/85	37/33	*χ*^2^ = 0.26	0.61
Smoker/non-smoker	52/143	17/53	*χ*^2^ = 0.15	0.70
Age (years)	35.60 (13.03)	39.74 (11.82)	*t* = 2.33	0.02
Education (years)	12.37 (3.21)	12.91 (2.56)	*t* = 1.28	0.20
BMI	23.43 (4.36)	23.92 (3.07)	*t* = 1.02	0.31
CPZ	266.31 (349.77)	NA	NA	NA
PANSS total score	72.36 (17.13)	NA	NA	NA
Working memory^a^	45.16 (11.43)	57.78 (7.65)	*F* = 78.19	<0.001
Processing speed^a^	44.96 (8.73)	57.09 (8.51)	*F* = 92.99	<0.001
Attention/vigilance^a^	43.64 (9.85)	56.81 (9.08)	*F* = 59.66	<0.001
Verbal learning^a^	46.98 (12.17)	57.41 (8.34)	*F* = 39.31	<0.001
Visual learning^a^	45.23 (10.56)	53.39 (8.47)	*F* = 37.73	<0.001
Reasoning and problem solving^a^	45.70 (10.70)	55.88 (7.81)	*F* = 69.88	<0.001
Social cognition^a^	46.20 (10.97)	53.22 (9.95)	*F* = 24.76	<0.001
MCCB composite score^a^	43.91 (10.21)	57.83 (7.93)	*F* = 103.64	<0.001
5-HI (ng/ml)^a^	8.57, 8.44 (2.79)	7.85, 7.73 (2.12)	*F* = 2.54	0.11
KYNA (ng/ml)^a^	6.42, 5.77 (3.36)	8.59, 8.14 (3.09)	*F* = 34.38	<0.001
QUIN (ng/ml)^a^	49.62, 44.71 (24.73)	54.18, 50.51 (16.55)	*F* = 5.63	0.02
5-HI/KYNA^a^	1.69, 1.36 (1.30)	1.01, 0.94 (0.40)	*F* = 33.61	<0.001
QUIN/KYNA^a^	8.67, 8.04 (3.8)	6.77, 6.36 (2.44)	*F* = 18.05	<0.001

Data reported as [mean (SD)], except for 5-HI, KYNA, QUIN, 5-HI/KYNA, and KYNA/QUIN as [mean, median (SD)].

*CPZ* chlorpromazine equivalent, *DBP* diastolic blood pressure, *5-HI* 5-hydroxyindole, *KYNA* kynurenic acid, *MCCB* MATRICS Consensus Cognitive Battery, *NA* not applicable, *PANSS* Positive and Negative Syndrome Scale, *QUIN* quinolinic acid, *SBP* systolic blood pressure.

^a^Statistics included sex and age as covariates.

### Clinical and cognitive assessments

Clinical symptoms were evaluated by the Positive and Negative Syndrome Scale (PANSS) in patients of schizophrenia by one of the three attending psychiatrists. The inter-rater intra-class correlation coefficient among the raters was above 0.80. Cognitive function was assessed using the validated Chinese version of the MATRICS Consensus Cognitive Battery (MCCB)^38–40^. The MCCB contains assessments of seven cognitive domains: Working Memory, Speed of Processing, Attention and Vigilance, Verbal Learning, Visual Learning, Reasoning and Problem Solving, and Social Cognition. Initial scores were converted to domain scores. Impaired working memory was evidenced a pervasive and the core of schizophrenia-related cognitive disability^41,42^; thus, our primary focus was on the working memory domain.

### Biochemistry

Blood samples were collected with BD Vacutainer serum tubes in the morning after overnight fasting and centrifuged immediately at 4 °C for 10 min at 3000 r.p.m. Serum was then aliquoted into separate tubes and stored at −80 °C. High-performance liquid chromatography was used to separate serum KYNA, 5-HI, and QUIN, and tandem mass spectrometry was then performed to quantify them using standard protocols. Details of the 5-HI assay, quality control, and validations were in Supplementary Material 1. Details of the KYNA and QUIN assays were described as previous reports^43–45^. The intra-trial coefficient of variations for high, median, and low quality were 1.2%, 2.7%, and 1.5% for 5-HI; 4.0%, 12.5%, and 8.8% for KYNA; and 4.1%, 6.2%, and 7.4% for QUIN, respectively. We further calculated the 5-HI/KYNA ratio and the QUIN/KYNA ratio to explore their putative agonistic/antagonistic effects on α7nAChR and NMDAR, respectively.

### Image processing

Structural images were collected in 153 schizophrenia patients and 65 HCs using a 3.0 Tesla Prisma MRI scanner (Siemens, Germany) and a 64-channel head coil at the MRI Research Center of the Beijing Huilongguan Hospital. Parameters of the three-dimensional magnetization prepared rapid acquisition gradient echo sequence were as follows: echo time = 2.98 ms, inversion time = 1100 ms, repetition time = 2530 ms, flip angle = 7°, field of view = 256 × 224 mm^2^, matrix size = 256 mm × 224 mm, and thickness/gap = 1/0 mm covering the whole brain. Participants used earplugs and foam pads to reduce magnetic machine noise and head movement, and who were reminded not to move their head during the procedure. A computer-connected monitor was acquired to detect head movement distance. The automated and validated segmentation were conducted by using FreeSurfer v5.3 (http://surfer.nmr.mgh.harvard.edu/)^46^. Thirty-four cortical gray matter regions in each hemisphere were extracted as regions of interest for statistical analysis according to the Desikan–Killiany atlas^47,48^.

### Statistical analysis

Normality of raw data was assessed with normal *Q*–*Q* plots and Shapiro–Wilkinson tests. Serum 5-HI, KYNA, QUIN levels, and 5-HI/KYNA ratio all deviated from normal distribution; all were normalized after natural logarithm (ln) or square root (sqrt) transformation. *T*-tests or *χ*^2^-test were used to compare the demographic data of patients with schizophrenia and HCs. Separate univariate analyses of covariance (ANCOVAs) were used to determine between-group differences in metabolites, MCCB and PANSS subdomain, and total scores, with sex and age as covariates. Multiple linear regression model was used to test whether working memory score was associated with the serum levels of 5-HI, KYNA, and QUIN in schizophrenia patients, adjusted for age and sex. Significant but opposite directions of associations of the metabolites with working memory would imply opposite roles. For those metabolites having significant opposite effects on working memory, we further divided patients into high- (concentration equal to or above the median) and low- (concentration below the median) level subgroups, and compared their working memory performance, adjusted for age and sex. Associations between cortical thickness of 68 cortical regions and serum levels of 5-HI, KYNA, and QUIN were further explored by multiple linear regression adjusted by age and sex in patients. These analyses were also repeated in HCs. The effects of smoking status and psychotropic medications on the three metabolites were also explored. Significance was set at *p* < 0.05 in all tests.

## Results

### Group differences

The summary demographics, clinical characteristics, and outcome variables were presented in Table 1. After controlling for sex and age, all seven domains and MCCB total score were lower in patients with schizophrenia compared to HCs (*F* = 24.76–78.19, *p* < 0.001). Patients with schizophrenia also had significantly lower serum KYNA (*F* = 34.38, *p* < 0.001) and QUIN (*F* = 5.63, *p* = 0.02) than in HCs, but 5-HI concentrations did not differ between groups (*F* = 2.54, *p* = 0.11). 5-HI/KYNA (*F* = 33.61, *p* < 0.001) and QUIN/KYNA (*F* = 18.05, *p* < 0.001) were significantly higher in patients with schizophrenia than in HCs. Data were also analyzed in medication-free patients. Medication-free patients showed significantly lower KYNA (5.36 ± 2.50 ng/mL vs. 7.31 ± 3.84 ng/mL; *F* = 11.87, *p* = 0.001) and QUIN (41.60 ± 12.26 ng/mL vs. 57.67 ± 30.89 ng/mL; *F* = 8.09, *p* = 0.01), but insignificant on 5-HI (8.63 ± 2.95 ng/mL vs. 8.44 ± 2.77 ng/mL; *F* = 0.35, *p* = 0.55) levels compared to patients on antipsychotic medications. As medication-free patients have even lower KYNA and QUIN levels than patients on antipsychotic medications, the low serum levels of KYNA and QUIN in schizophrenia are unlikely due to antipsychotic medication effects.

### Relationship of working memory with 5-HI, KYNA, and QUIN

The multiple linear regression model using the three metabolites as predictors was significant (*F* = 3.18, *p* = 0.01), wherein working memory was significantly predicted by higher level of 5-HI (*t* = 2.10, *p* = 0.04) and lower KYNA (*t* = −2.01, *p* = 0.05) (Table 2). The results were not statistically significant, although 5-HI (*t* = 0.94, *p* = 0.35) and KYNA (*t* = −1.48, *p* = 0.14) had the same trend in the medication-free group, similar to findings in the total-patient group. One possible reason is that each divided group may have lower statistic power than the total-patient group. The results were still significant (*F* = 10.11, *p* < 0.001) when we added smoke and CPZ equivalent in the model, and 5-HI (*t* = 2.18, *p* = 0.03) and KYNA (*t* = −2.24, *p* = 0.03) have the same opposite trends as before. The model was insignificant in HCs (model *p* = 0.83). We also explored other MCCB domains and total score but found no significant results in either patients or controls (Supplementary Table 2).

**Table 2:** Multiple regression analysis results of serum levels of 5-HI, KYNA, and QUIN on working memory score.

	Schizophrenia			Healthy controls		
	Standardized *β*	*t*	*p*	Standardized *β*	*t*	*P*
5-HI	0.15	2.10	**0.04**	−0.02	−0.19	0.85
KYNA	−0.18	−2.01	**0.05**	−0.02	−0.14	0.89
QUIN	0.06	0.68	0.49	0.11	0.75	0.46
Sex	0.16	2.04	**0.04**	0.16	1.18	0.24
Age	−0.02	−0.26	0.80	−0.10	−0.10	0.92
Model	***F*** **=** **3.18**, ***p*** **=** **0.01**			*F* = 0.42, *p* = 0.83		

*5-HI* 5-hydroxyindole, *KYNA* kynurenic acid, *QUIN* uinolinic acid.

Bold values indicates statistical significance at *p* < 0.05.

**Fig. 2:**
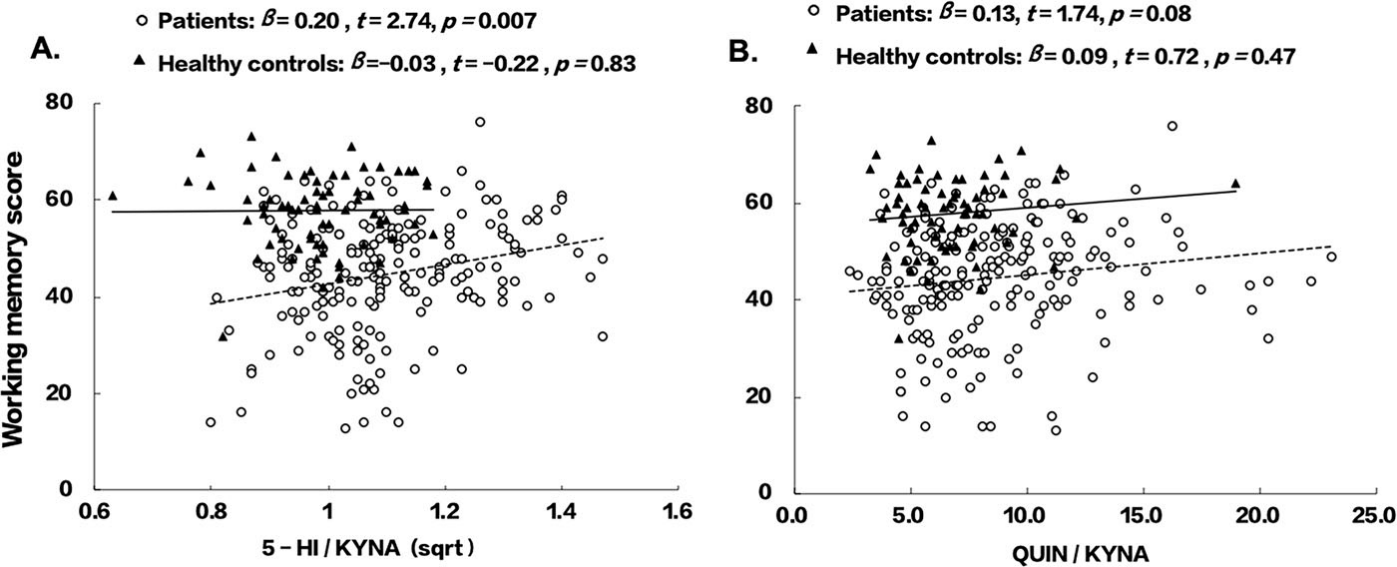
Relationship between working memory performance and 5-HI/KYNA or QUIN/KYNA. **A** Relationship of working memory with 5-HI/KYNA. **B** Relationship of working memory with QUIN/KYNA.

The relationships of working memory with 5-HI/KYNA and QUIN/KYNA were also explored. 5-HI/KYNA was positively associated with working memory (*t* = 2.74, *p* = 0.007) in patients, but not in HCs (Fig. 2). There was no significant correlation with QUIN/KYNA in either patients or controls.

**Fig. 3:**
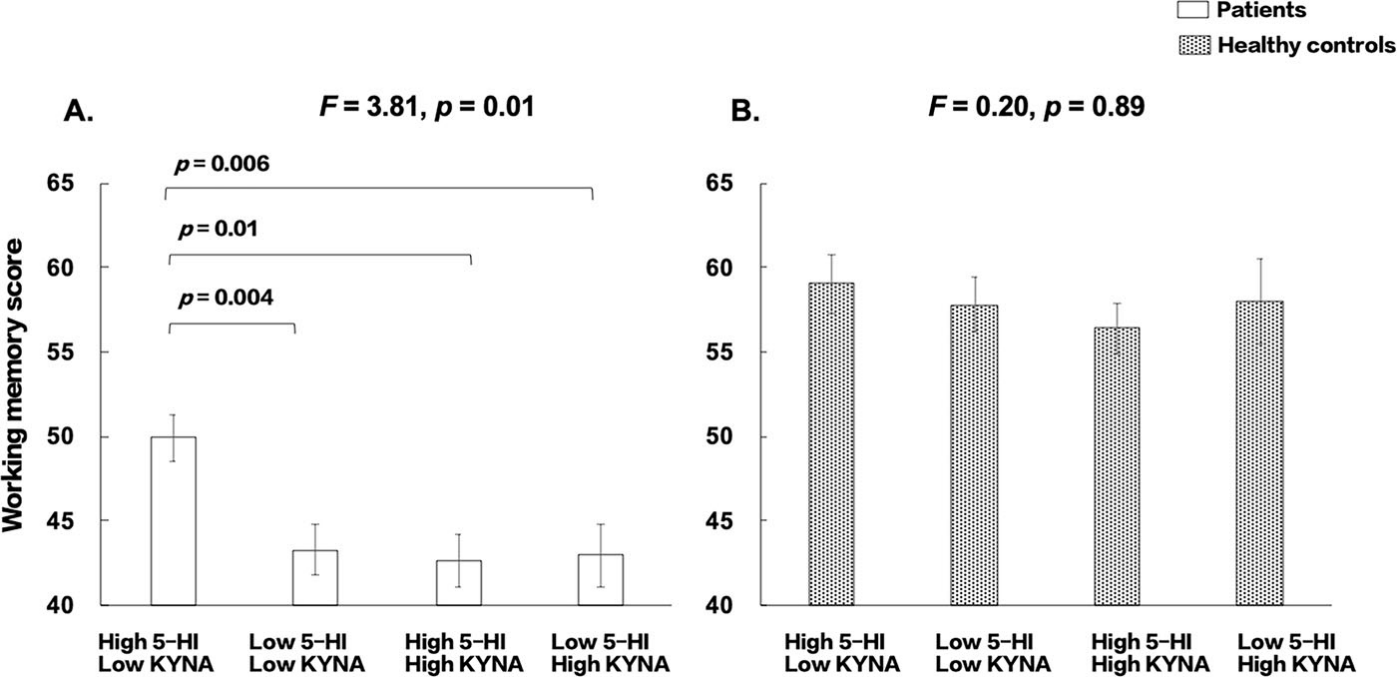
Comparisons of working memory performance in subgroups with 5-HI and KYNA levels by medium splits adjusting for age and sex. **A** Patients with schizophrenia. **B** Healthy controls.

We further divided the patients into high- and low-level in four subgroups based on medium splits for 5-HI and KYNA: high 5-HI/low KYNA, low 5-HI/low KYNA, high 5-HI/high KYNA, and high 5-HI/low KYNA (Fig. 3). The overall ANCOVA was significant (*F* = 3.81, *p* = 0.01). Post-hoc tests showed that patients with high 5-HI and low KYNA had the best working memory performance compared with the other three subgroups (*p* = 0.01–0.004); however, the other three subgroups did not significantly differ. The model was not significant in HCs (*F* = 0.20, *p* = 0.89).

### Relationship of cortical thickness with 5-HI, KYNA, and QUIN

**Fig. 4:**
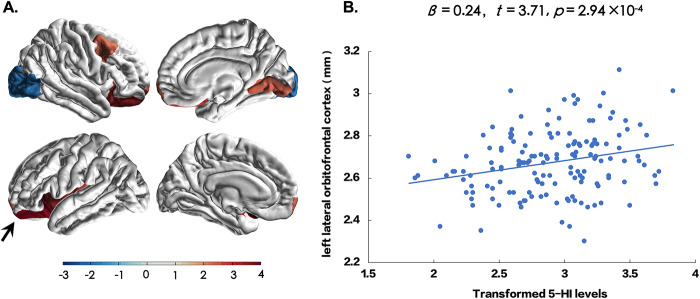
The relationship of serum levels of 5-HI with cortical thickness in patients with schizophrenia. The left and right frontal poles, left insula, right caudal-middle frontal, left and right lateral orbitofrontal, and right lingual areas were positively and significantly associated with 5-HI at *p* = 0.03–0.0003, and the right lateral occipital region was inversely associated with 5-HI at *p* = 0.05, but only the lateral orbitofrontal cortex (arrow) was significant after correction for multiple comparisons. **A** Cortical regions were colored according to *t*-scores by multiple linear regression. The color bar represents *t*-scores. **B** Scatter plot between 5-HI and the left lateral orbitofrontal cortex (arrow in **A**) in patients with schizophrenia.

Multiple cortical regions showed significantly reduced cortical thickness in patients compared to HCs (Supplementary Table 3). We next explored the associations of 5-HI, KYNA, and QUIN with thickness of 68 cortical regions after controlling for sex and age. Only the left lateral orbitofrontal cortex (LOFC) showed significantly positive association with the 5-HI levels (*t* = 3.71, *p* = 2.94 × 10^−4^) after Bonferroni correction for multiple comparisons (0.05/68 = 7.35 × 10^−4^) (Fig. 4). However, we also observed nominally significant (*p* < 0.05 uncorrected) positive associations between 5-HI and the left and right frontal poles, left insula, right caudal-middle frontal, right lateral orbitofrontal, and right lingual areas in patients with schizophrenia (*p* = 0.03–0.004), whereas the right lateral occipital region had a nominally negative association with 5-HI (*p* = 0.05) (Supplementary Table 4). In medication-free patients, 5-HI was still correlated with LOFC thickness (*r* = 0.39, *p* = 1.4 × 10^−4^). There were no significant associations between cortical thickness and 5-HI in HCs. No significant associations between cortical thickness and KYNA, QUIN, or their ratios were found in patients or controls after correction for multiple comparisons (Supplementary Table 4).

The left LOFC thickness was positively associated with working memory (*t* = 2.68, *p* = 0.008). The left LOFC thickness was also associated with verbal learning (*t* = 4.27, *p* < 0.001), social cognition (*t* = 3.05, *p* = 0.003), and reasoning and problem solving (*t* = 2.47, *p* = 0.02) (Supplementary Table 5).

### Relationship of tryptophan metabolites with sex, age, smoking, blood pressure, heart rate, and medication

After controlling for sex and age, CPZ was negatively correlated with 5-HI (*r* = −0.24, *p* = 0.04), but not with KYNA or QUIN (both *p* > 0.05). Males had higher 5-HI (8.70 ± 2.58 ng/mL), KYNA (7.65 ± 3.65 ng/mL), and QUIN (54.34 ± 26.23 ng/mL) concentrations than females (7.93 ± 2.75, 5.86 ± 2.66, and 45.45 ± 15.29 ng/mL, respectively) (*F* = 6.19, *p* = 0.01; *F* = 25.68, *p* < 0.001; *F* = 10.40, *p* = 0.001, respectively), but sex × diagnosis interactions were insignificant (all *p* > 0.20). Non-smokers had significantly lower KYNA concentrations than smokers (6.57 ± 3.27 ng/mL vs. 7.55 ± 3.45 ng/mL; *F* = 6.67, *p* = 0.01), but smoking × diagnosis interaction was insignificant (*p* = 0.36). Smokers and non-smokers did not significantly differ in 5-HI (*p* = 0.66) or QUIN (*p* = 0.31) levels, nor were the interactions of smoking with diagnosis (*p* = 0.37 and 0.15, respectively). Age was significantly correlated with KYNA (*r* = 0.13, *p* = 0.03) and QUIN (*r* = 0.18, *p* = 0.001) concentrations, but not with 5-HI (*p* = 0.06). We examined systolic and diastolic blood pressure, and heart rate in relation to KYNA, 5-HI, and QUIN, and found that there were insignificant correlations in either patients or HCs (all *p*’s > 0.05).

## Discussion

This study examined the effects of 5-HI, KYNA, and QUIN on working memory and cortical structures, based on preclinical evidence of their effects on α7nAChR and NMDAR. In patients with schizophrenia, KYNA and QUIN, but not 5-HI, were lower than in HCs. Working memory was significantly influenced by 5-HI and KYNA but in opposite direction in the patients. Patients exhibiting high 5-HI and low KYNA concentrations had better working memory performance than the other subgroups. Finally, 5-HI level was positively associated with cortical thickness of the left orbitofrontal cortex, which was significantly associated with working memory performance in the patients.

5-HI is a relatively potent tryptophan metabolite that increases glutamate release and the function of GABA interneurons^21^, and in high dose can cause convulsion^49^. 5-HI precursor indole is transformed from tryptophan by tryptophanase in indole-producing bacteria of human gut^50,51^. Diverse oxygenase could degrade indole to indole derivatives^52,53^ such as 5-HI. The formation of 5-HI can be observed by incubating rat liver homogenates with its direct precursor indole, supporting an endogenous production of 5-HI^21^. In rats with hepatic encephalopathy, indole produced by gut bacteria is absorbed and metabolized into several metabolites including 5-HI, which may accumulate in the blood and brain^17,21,54^. Earlier clinical research has suggested that blood 5-HI may provide treatment effect monitoring for hyperactive behaviors, provided some initial support for its potential clinical utility^55^. Previous rodent studies indicated that 5-HI activates both presynaptic and postsynaptic α7nAChR to mediate glutamate release^18,19,22^, which may facilitate working memory. In addition, 5-HI increases acetylcholine (ACh) efficacy though Ca^2+^ currents, indicating that 5-HI and ACh may cooperate to influence α7nAChR^18^. Regardless of whether our finding here is related to α7nAChR, the data provided the first evidence in humans, supporting the hypothesis that 5-HI may facilitate working memory performance in patients with schizophrenia.

Reduced serum KYNA concentration in schizophrenia was highly replicable in several recent reports^56–58^, which were not consistent with several earlier studies^59,60^. Our results are also inconsistent with studies showing elevated KYNA in the post-mortem brains of patients with schizophrenia^4,45,61^. As KYNA normally does not pass the BBB^62^, it is unclear whether the observed inverse correlation between serum KYNA levels and working memory is due to these brain mechanisms; as such, the KYNA-related results should be viewed with caution. However, impaired BBB has been proposed in patients with schizophrenia^63,64^, which may explain this significant correlation only in patients but not in controls. Moreover, evidences showed peripheral kynurenine can pass through BBB^62,65^ and may influence brain functions^2,66^. Preclinical studies have demonstrated that KYNA negatively affects α7nAChR-dependent presynaptic mechanisms in the prefrontal cortex and disrupts local GABAergic synaptic signaling^67^. Knocking out kynurenine aminotransferase II that decreases endogenous brain KYNA can increase working memory performance^68,69^. At high concentrations, KYNA is a competitive antagonist of NMDAR^70,71^; however, at low concentrations, KYNA has a more potent inhibitory effect on α7nAChR activation than on NMDAR^4^. Furthermore, reducing KYNA increases 5-HI-dependent activation of α7nAChR^19^. Therefore, our data appear as corroborating these preclinical data on the opposing 5-HI vs. KYNA effects, showing that patients having high 5-HI and low KYNA was associated with the best working memory.

However, why patients have reduced serum KYNA but there is an inverse correlation between KYNA and working memory is difficult to interpret. Similar to our finding, significantly low peripheral serum kynurenine and/or KYNA levels have been found in diverse cohorts of patients^56–58,72^. Meanwhile, higher KYNA in the brain is thought to impair cognitive functions^2^, which appears consistent with our finding of an inverse correlation between peripheral KYNA and working memory. We believe that the difficulty to explain the seemingly contradictory findings is in part due to the lack of a good explanation on why peripheral kynurenine and/or KYNA are reduced in schizophrenia, while brain KYNA appears increased in schizophrenia, compared with that in HCs. Further complicating the issue is that about 80% of kynurenine and KYNA in the blood were bound to albumin or other circulating binding proteins^62,73^, which may lead to differential availability of free kynurenine and KYNA, and our study is limited by not measuring specifically the free serum kynurenine or KYNA. Basic neuroscience effort to simultaneously assessing central and peripheral kynurenine and KYNA, while invasively studying the potential mechanism underlying the central–peripheral metabolite relationships may be needed.

We observed that serum QUIN concentration was significantly lower in patients with schizophrenia than in HCs. Previous studies attempted to identify QUIN-related abnormalities in schizophrenia but largely failed to show a significant differences in QUIN levels between patients and controls in post-mortem brain tissues^45^, blood^57^, or CSF^74^. However, QUIN normally also does not pass the BBB^62^ and our analysis did not show significant relationship between QUIN and working memory or other clinical and cognitive measures.

An association between lower 5-HI to thinner cortical thickness of the LOFC in patients is intriguing. Previous researches indicated that the orbitofrontal cortex supports working memory^75^, specifically encoding gustatory^75^, emotional^76^, and abstract information^77^. The LOFC is particularly important for reward learning^78^, a process closely related to working memory^79^. We consider the finding linking 5-HI to the thickness of the LOFC a further supportive evidence of a potential cognition enhancement effect of this metabolite in patients. Also some evidences showed relationships between kynurenine and subcortical volumes in mental disorders that KYNA/3-hydroxykynurenine were positively correlated to the hippocampal volume in bipolar disorder^80^ and negatively correlated with the left hippocampal activity in major depressive disorder^81^. We further explored the associations between subcortical volumes, and KYNA and KYNA/QUIN. However, there was no significant results in patients or in HCs (Supplementary Material 6).

The study has a number of limitations. We did not test whether the effects of these metabolites occurred through α7nAChR, NMDAR, or other receptor mechanisms in the brain, although 5-HI is lipophilic and may be BBB permeable^20^. However, KYNA and QUIN do not normally pass the BBB, making the blood-based finding or the lack thereof difficult to explain. We also did not measure the 5-HI precursor indole (Fig. 1) to rule out its contributions to the observations here. However, indole itself was thought not to interact with GABAergic or ionotropic glutamate receptors^17^. Furthermore, we measured 5-hydroxyoxindole (5-HOI), another metabolite from the indole and oxindole branch (Fig. 1), to confirm that the 5-HI results reported here is distinct from 5-HOI (Supplementary Material 7). We also did not measure the gut flora that possibly influence the concentration of indole and further related to 5-HI level. The inflammatory markers were not tested in the present study, which may limit the interpretation of relationship between metabolites, as high inflammatory activity can lead to elevations of kynurenine and KYNA^82–84^. We also did not measure diet and physical activities that may have an effect on peripheral levels of KYNA^85,86^, cortical thickness^87^, and cognition^88^, which is another limitation. Despite these limitations, the observed pattern of higher working memory in patients with high 5-HI and low KYNA appears consistent with the directions of their mechanism of action^19^.

In summary, our findings suggest that there appears multiple potential mechanisms by which the tryptophan pathway is relevant to the cognitive performance in schizophrenia, including the indole branch whose relationship to schizophrenia has not been previously studied. Therefore, the finding that 5-HI and KYNA may have opposing effects on working memory among patients with schizophrenia is new and is consistent with preclinical evidences on their counteractive mechanisms. Our data may provide new insight into potential targets in the tryptophan pathway in our effort to develop novel therapeutic strategy for treating working memory and other cognitive impairment in schizophrenia.

## Supplementary information

Supplemental material
