# Supplementary material for: Effects of neuroactive metabolites of the tryptophan pathway on working memory and cortical thickness in schizophrenia
Source: Transl Psychiatry. 2021 Apr 1;11:198. doi: 10.1038/s41398-021-01311-z (PMC8016899; doi:10.1038/s41398-021-01311-z)
Supplement: Supplementary file 1 — Supplemental material [file 41398_2021_1311_MOESM1_ESM.docx]

**Effects of Neuroactive Metabolites of the Tryptophan Pathway on Working Memory and Cortical Thickness in Schizophrenia**

**Supplementary material 1.** **LC-MS/MS assay for the quantification of 5-HI**

**1.1 Materials and Methods**

**1.1.1 Chemicals and reagents**

5-hydroxyindole (5-HI, purity 98.5%) was obtained from the Macklin reagent company (Shanghai, China). 5-hydroxytryptamine-d4 HCl (5-HT-d4, purity >98%) was purchased from ISOREAG standards (Shanghai, China). Formic acid and methanol were acquired from Merck (Darmstadt, Germany). Purified water was obtained from A.S. Watson Group (Hongkong, China).

**1.1.2 Chromatographic and mass spectrometric conditions**

A Waters Acquity UPLC I-class system (Waters, Shanghai) equipped with Waters Xevo TQS IVD system (Waters, Shanghai) with ESI source was used. The chromatographic separation was achieved on a Waters Acquity UPLC HSS T3 (2.1 × 50 mm, 1.8 μm) column with a column temperature of 40℃. A gradient elution with a flow rate of 0.3 mL/min using 0.1% formic acid (FA) in water as solvent A and 0.1% formic acid (FA) in methanol as solvent B was performed. The elution program was set as follows: 0-0.5 min 5% B, 0.5-1 min 5%-80% B, 1-2 min 80% B, 2-2.5 min 80%-5% B, 2.5-3.5 min 5% B. The sampler chamber temperature kept at 10℃.

**1.1.3 Mass Spectrometric parameters**

The mass detection and quantification were performed in a positive ion mode. The mass spectrometer working parameters were optimized as follows: capillary voltage of 3.5 kV, ion source temperature of 150℃, desolvation temperature of 550℃, desolvation gas flow of 800 L/Hr and cone gas flow of 150 L/Hr. Multipole reaction monitoring (MRM) mode was used for the analysis with mass transition parameters as follows:

**Supplementary table 1.1. Mass transition parameters**

| **Analytes** | **parent(m/z)** | **daughter(m/z)** | **Dwell(s)** | **cone(V)** | **collision(V)** |
| --- | --- | --- | --- | --- | --- |
| **5-HI**  **(Qualitative analysis)** | 134.16 | 106.41 | 0.025 | 20 | 16 |
| **5-HI**  **（Quantitative analysis）** | 134.16 | 116.18 | 0.025 | 20 | 16 |
| **5-HT-d4** | 181.16 | 163.96 | 0.025 | 10 | 8 |

**1.1.4 Calibrators and quality controls**

Standard stock solution of 5-HI and 5-HT-d4 at a concentration of 1.0 mg/ml was separately prepared in 60% methanol with 4 mg/mL ascorbic acid as protective agent. The working solutions were prepared by a series of dilution with 80% methanol. All solutions were stored at –20°C.

An aliquot of 10 µL different working solutions was spiked with 90 µL 50% methanol/water aliquot separately and vortexed for 1 min to prepare calibration standard samples at different concentration levels. The calibrator concentrations were 11.13, 27.825, 55.65, 111.3, 278.25, 556.5, 1113 ng/mL for 5-HI. The QC samples were prepared as the same procedures to give different concentrations of 5-HI at 111.3, 278.25, 556.5 ng/mL.

**1.1.5 Preparation of samples**

An aliquot of 300 μL internal standard (IS) solution (50 ng/mL 5-HT-d4 in methanol) was added to 100 μL sample and vortexed for 3 min. The mixture was then centrifuged at 4℃ and 15000 rpm for 10 min. 100 μL supernatant was then transferred to a clean vial with the addition of 100 μL pure water and vortexed for 1 min. The mixture was then followed by centrifugation at 4℃ and 15000 rpm for 5 min. The 100 μL supernatant was finally injected into the LC-MS/MS system.

**1.1.6 Validation of the methods**

The established method was validated for specificity, linearity, accuracy and precision.

1.1.6.1 Selectivity.

Selectivity of the method was assessed by observing the interference at the retention time of analysts and IS in plasma sample.

1.1.6.2 Calibration curve linearity.

The calibration curve was constructed by plotting the peak area ratios of each analyte/IS versus its nominal concentrations, using a linear regression.

1.1.6.3 Accuracy and precision.

The replicates (n=5) of QC samples were analyzed to determine the precision and accuracy of the method. The precision is expressed by CV between the replicate measurements. Accuracy is defined as relative error (RE) which is calculated using the formula RE% = [(measured value−theoretical value)/ theoretical value] ×100%.

**1.2 Results**

**1.2.1 Selectivity**

The MRM chromatograms of analytes were presented in Figure 1.1. The peak shapes of all the analytes were of good quality for assay. The total ion chromatography of plasma sample, the blank 50% methanol solution and QC sample were shown in Figure 1.1. No significant chromatographic peak area interference was observed either at the retention time of analytes or IS in blank 50% methanol solution.

**Supplementary figure 1.1. The MRM chromatograms of 5-HI in the blank 50% methanol solution (A), QC sample (B) and serum sample(C).**


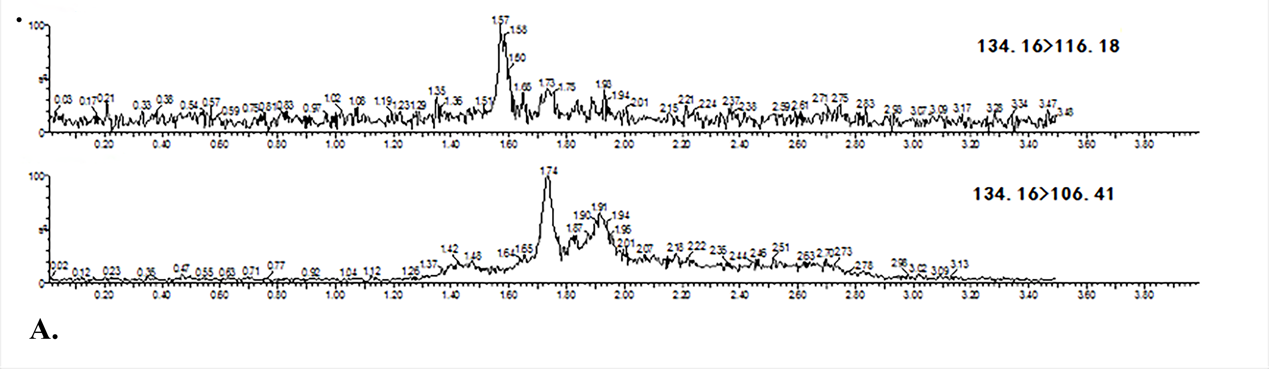


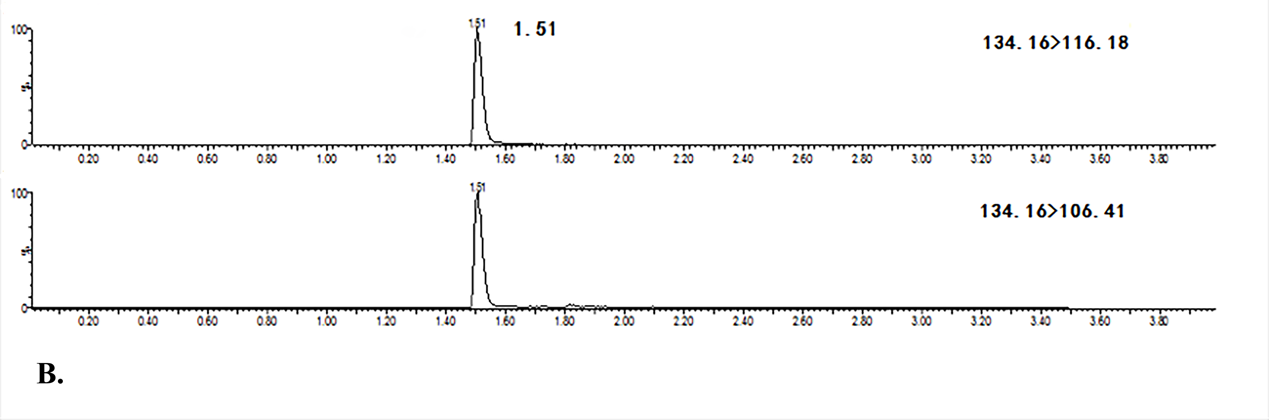


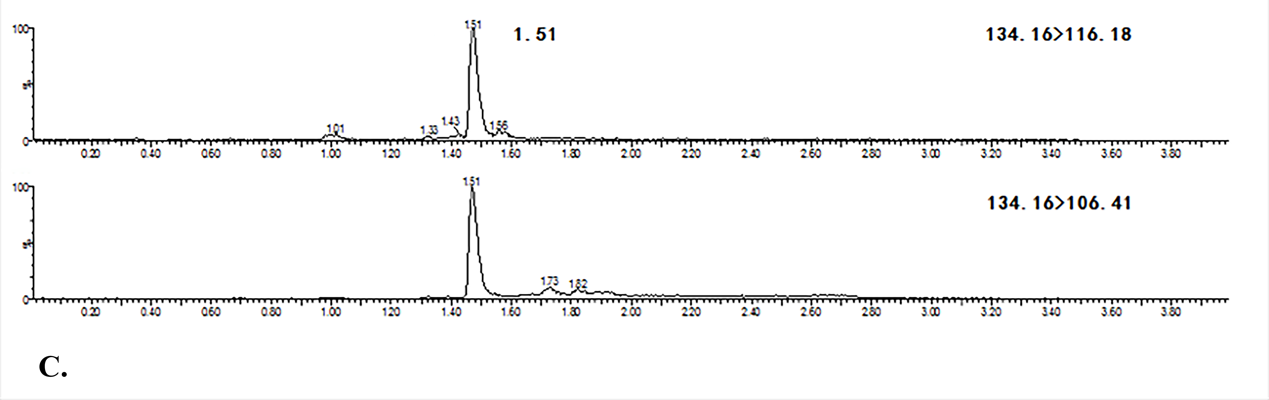


**1.2.2 Calibration curve linearity.**

The calibration curves are showed in Figure 1.2. all calibration curves exhibited good linearity with correlation coefficient (r^2^) within the range of 0.9902–0.9998.

**Supplementary figure 1.2. The calibration curve for 5-HI.**


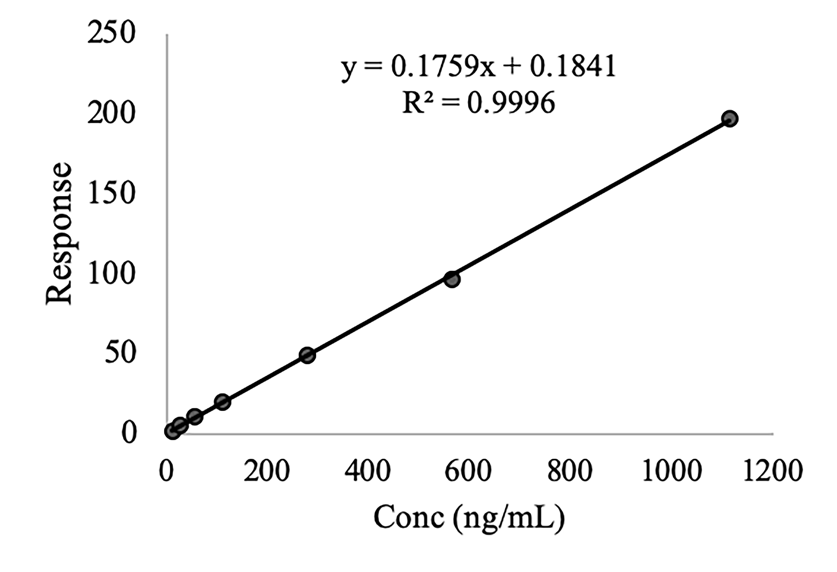


**1.2.3 Accuracy and precision.**

As presented in the Table 1.2, the precision (CV) ranged between 1.16% and 2.71 %, and accuracy (RE) ranged between 0.35% and 6.79 %, which were acceptable for biological analysis.

**Supplementary table 1.2. The precision and accuracy of the method.**

| **Analytes** | **Nominal Conc (ng/mL)** | **CV（%）** | **RE（%）** |
| --- | --- | --- | --- |
| **5-HI** | 111.3 | 1.45 | 3.66 |
|  | 278.3 | 2.71 | 0.35 |
|  | 565.5 | 1.16 | 6.79 |

**Supplementary table 2.** **Multiple regression analysis results of serum levels of 5-HI, KYNA and QUIN on MCCB scores.**

|  |  | Model | 5-HI | KYNA | QUIN | Sex | Age |
| --- | --- | --- | --- | --- | --- | --- | --- |
|  |  | *F* Value | *t* Value | *t* Value | *t* Value | *t* Value | *t* Value |
| Group | Domain | *p* Value | *p* Value | *p* Value | *p* Value | *p* Value | *P* Value |
| Schizophrenia | Working memory | 3.18 | 2.10 | -2.01 | 0.68 | 2.04 | -0.26 |
|  |  | **0.01** | **0.04** | **0.05** | 0.49 | **0.04** | 0.80 |
|  | Processing speed | 0.28 | -0.25 | -0.01 | 0.85 | 0.83 | -0.23 |
|  |  | 0.92 | 0.80 | 1.00 | 0.40 | 0.41 | 0.82 |
|  | Attention/vigilance | 1.31 | 1.08 | 0.03 | 0.49 | 1.02 | 1.97 |
|  |  | 0.26 | 0.28 | 0.98 | 0.63 | 0.31 | 0.05 |
|  | Verbal learning | 1.98 | 1.60 | -0.38 | -0.16 | 2.08 | 1.63 |
|  |  | 0.08 | 0.11 | 0.71 | 0.87 | 0.04 | 0.10 |
|  | Visual learning | 2.28 | -0.04 | 0.33 | 1.22 | 3.10 | -1.08 |
|  |  | **0.05** | 0.97 | 0.74 | 0.22 | **0.002** | 0.28 |
|  | Reasoning and problem solving | 5.78 | 0.91 | -0.12 | 0.61 | 0.78 | -5.10 |
|  |  | **<0.001** | 0.37 | 0.91 | 0.54 | 0.43 | **<0.001** |
|  | Social cognition | 0.77 | -0.01 | -0.24 | 0.48 | 1.28 | -1.41 |
|  |  | 0.57 | 1.00 | 0.81 | 0.64 | 0.20 | 0.16 |
|  | MCCB total score | 1.79 | 1.30 | -1.11 | 0.97 | 1.95 | -0.77 |
|  |  | 0.12 | 0.20 | 0.27 | 0.33 | 0.05 | 0.44 |
| Healthy controls | Working memory | 0.42 | -0.19 | -0.14 | 0.75 | 1.18 | -0.10 |
|  |  | 0.83 | 0.85 | 0.89 | 0.46 | 0.24 | 0.92 |
|  | Processing speed | 0.32 | -0.41 | -1.02 | 0.64 | -0.59 | 0.35 |
|  |  | 0.90 | 0.68 | 0.31 | 0.52 | 0.56 | 0.73 |
|  | Attention/vigilance | 0.53 | 0.18 | -1.10 | 1.30 | -0.30 | 0.62 |
|  |  | 0.75 | 0.86 | 0.28 | 0.20 | 0.76 | 0.54 |
|  | Verbal learning | 1.19 | -2.13 | 0.76 | 0.31 | 0.61 | 0.11 |
|  |  | 0.32 | 0.04 | 0.45 | 0.75 | 0.55 | 0.92 |
|  | Visual learning | 0.45 | 0.55 | -0.56 | 0.10 | -0.29 | -1.00 |
|  |  | 0.81 | 0.59 | 0.58 | 0.93 | 0.77 | 0.32 |
|  | Reasoning and problem solving | 0.73 | -0.01 | -1.66 | 0.57 | 0.002 | -0.52 |
|  |  | 0.60 | 0.99 | 0.10 | 0.57 | 1.00 | 0.60 |
|  | Social cognition | 1.03 | -1.03 | 0.77 | -1.15 | -1.37 | -0.26 |
|  |  | 0.41 | 0.31 | 0.45 | 0.26 | 0.18 | 0.80 |
|  | MCCB total score | 0.11 | -0.28 | -0.59 | 0.26 | -0.48 | -0.07 |
|  |  | 0.99 | 0.78 | 0.56 | 0.80 | 0.64 | 0.94 |

Abbreviations: 5-HI: 5-hydroxyindole; KYNA: Kynurenic acid; QUIN: Quinolinic acid.

**Supplementary table 3.** **A comparison of cortical thicknesses between schizophrenia patients and healthy controls adjusting for sex and age**

| Regions | Schizophrenia  (n=153) | Healthy controls  (n=65) | *F* Value | *p* Value |
| --- | --- | --- | --- | --- |
| L_bankssts | 2.55(0.02) | 2.61(0.13) | 16.17 | <0.001 |
| L_caudalanteriorcingulate | 2.68(0.25) | 2.78(0.24) | 15.00 | <0.001 |
| L_caudalmiddlefrontal | 2.62(0.12) | 2.65(0.11) | 3.12 | 0.08 |
| L_cuneus | 1.90(0.13) | 1.88(0.12) | 0.20 | 0.66 |
| L_entorhinal | 3.47(0.26) | 3.52(0.22) | 1.48 | 0.23 |
| L_fusiform | 2.79(0.12) | 2.85(0.09) | 20.88 | <0.001 |
| L_inferiorparietal | 2.49(0.13) | 2.57(0.09) | 25.38 | <0.001 |
| L_inferiortemporal | 2.85(0.14) | 2.92(0.11) | 20.36 | <0.001 |
| L_isthmuscingulate | 2.35(0.21) | 2.40(0.15) | 4.81 | 0.03 |
| L_lateraloccipital | 2.20(0.13) | 2.29(0.11) | 18.97 | <0.001 |
| L_lateralorbitofrontal | 2.67(0.15) | 2.69(0.11) | 4.52 | 0.04 |
| L_lingual | 2.02(0.14) | 1.97(0.12) | 2.32 | 0.13 |
| L_medialorbitofrontal | 2.54(0.15) | 2.58(0.10) | 10.03 | 0.002 |
| L_middletemporal | 2.90(0.15) | 2.96(0.10) | 18.54 | <0.001 |
| L_parahippocampal | 2.64(0.29) | 2.67(0.24) | 1.53 | 0.22 |
| L_paracentral | 2.54(0.13) | 2.55(0.13) | 1.09 | 0.30 |
| L_parsopercularis | 2.62(0.14) | 2.67(0.11) | 13.58 | <0.001 |
| L_parsorbitalis | 2.69(0.19) | 2.73(0.16) | 6.38 | 0.01 |
| L_parstriangularis | 2.49(0.13) | 2.51(0.11) | 3.81 | 0.05 |
| L_pericalcarine | 1.63(0.15) | 1.59(0.14) | 1.65 | 0.20 |
| L_postcentral | 2.15(0.11) | 2.20(0.14) | 9.26 | 0.003 |
| L_posteriorcingulate | 2.52(0.17) | 2.55(0.14) | 5.88 | 0.02 |
| L_precentral | 2.68(0.13) | 2.70(0.13) | 3.01 | 0.08 |
| L_precuneus | 2.44(0.11) | 2.47(0.10) | 7.72 | 0.006 |
| L_rostralanteriorcingulate | 2.83(0.22) | 2.91(0.20) | 15.47 | <0.001 |
| L_rostralmiddlefrontal | 2.42(0.11) | 2.44(0.09) | 6.45 | 0.01 |
| L_superiorfrontal | 2.77(0.13) | 2.82(0.11) | 19.59 | <0.001 |
| L_superiorparietal | 2.27(0.13) | 2.35(0.09) | 19.78 | <0.001 |
| L_superiortemporal | 2.80(0.15) | 2.87(0.12) | 25.81 | <0.001 |
| L_supramarginal | 2.56(0.13) | 2.65(0.11) | 36.73 | <0.001 |
| L_frontalpole | 2.82(0.24) | 2.84(0.19) | 1.77 | 0.18 |
| L_temporalpole | 3.66(0.27) | 3.73(0.28) | 3.93 | 0.05 |
| L_transversetemporal | 2.42(0.21) | 2.41(0.18) | 0.54 | 0.46 |
| L_insula | 3.0(0.21) | 2.98(0.17) | 0.64 | 0.43 |
| R_bankssts | 2.63(0.15) | 2.65(0.16) | 3.30 | 0.07 |
| R_caudalanteriorcingulate | 2.56(0.22) | 2.67(0.21) | 16.80 | <0.001 |
| R_caudalmiddlefrontal | 2.58(0.12) | 2.63(0.11) | 12.83 | <0.001 |
| R_cuneus | 1.96(0.13) | 1.95(0.12) | <0.001 | 1.0 |
| R_entorhinal | 3.60(0.31) | 3.66(0.27) | 1.74 | 0.19 |
| R_fusiform | 2.80(0.13) | 2.87(0.10) | 25.50 | <0.001 |
| R_inferiorparietal | 2.50(0.14) | 2.60(0.10) | 33.80 | <0.001 |
| R_inferiortemporal | 2.82(0.13) | 2.89(0.09) | 18.22 | <0.001 |
| R_isthmuscingulate | 2.31(0.19) | 2.35(0.16) | 6.44 | 0.01 |
| R_lateraloccipital | 2.26(0.13) | 2.34(0.12) | 15.00 | <0.001 |
| R_lateralorbitofrontal | 2.65(0.16) | 2.67(0.11) | 6.16 | 0.01 |
| R_lingual | 2.04(0.13) | 2.01(0.12) | 0.79 | 0.38 |
| R_medialorbitofrontal | 2.53(0.15) | 2.56(0.12) | 5.58 | 0.02 |
| R_middletemporal | 2.91(0.14) | 2.97(0.10) | 18.61 | <0.001 |
| R_parahippocampal | 2.63(0.24) | 2.71(0.22) | 9.83 | 0.002 |
| R_paracentral | 2.57(0.14) | 2.60(0.13) | 3.12 | 0.08 |
| R_parsopercularis | 2.62(0.15) | 2.67(0.11) | 16.41 | <0.001 |
| R_parsorbitalis | 2.71(0.19) | 2.73(0.15) | 3.92 | 0.05 |
| R_parstriangularis | 2.49(0.14) | 2.54(0.13) | 16.19 | <0.001 |
| R_pericalcarine | 1.65(0.14) | 1.59(0.14) | 7.67 | 0.006 |
| R_postcentral | 2.15(0.11) | 2.18(0.09) | 5.25 | 0.02 |
| R_posteriorcingulate | 2.48(0.16) | 2.51(0.16) | 5.93 | 0.02 |
| R_precentral | 2.63(0.13) | 2.66(0.12) | 4.58 | 0.03 |
| R_precuneus | 2.44(0.11) | 2.51(0.09) | 24.97 | <0.001 |
| R_rostralanteriorcingulate | 2.90(0.21) | 2.92(0.20) | 3.33 | 0.07 |
| R_rostralmiddlefrontal | 2.39(0.12) | 2.40(0.09) | 2.41 | 0.12 |
| R_superiorfrontal | 2.74(0.13) | 2.80(0.11) | 19.62 | <0.001 |
| R_superiorparietal | 2.24(0.14) | 2.34(0.10) | 24.22 | <0.001 |
| R_superiortemporal | 2.84(0.15) | 2.90(0.13) | 21.71 | <0.001 |
| R_supramarginal | 2.54(0.13) | 2.64(0.11) | 37.11 | <0.001 |
| R_frontalpole | 2.76(0.23) | 2.80(0.20) | 3.72 | 0.06 |
| R_temporalpole | 3.75(0.32) | 3.79(0.29) | 1.81 | 0.18 |
| R_transversetemporal | 2.47(0.19) | 2.50(0.21) | 3.18 | 0.08 |
| R_insula | 2.99(0.18) | 3.03(0.15) | 9.87 | 0.002 |

Variance reported as mean (standard deviation). Abbreviations: 5-HI: 5-hydroxyindole; KYNA: Kynurenic acid; QUIN: Quinolinic acid; L: left; R: right.

**Supplementary table 4. Multiple regression analysis results of serum levels of 5-HI, KYNA and QUIN on** **cortical thickness controlled for sex and age**

|  | Schizophrenia | | | Healthy controls | | |
| --- | --- | --- | --- | --- | --- | --- |
| Regions | 5-HI | KYNA | QUIN | 5-HI | KYNA | QUIN |
| L_bankssts | 0.21(0.84) | -0.94(0.35) | 0.07(0.95) | 0.02(0.98) | -0.53(0.60) | 0.98(0.33) |
| L_caudalanteriorcingulate | -0.33(0.74) | 1.22(0.23) | -0.02(0.98) | -0.08(0.94) | 0.72(0.47) | -0.38(0.70) |
| L_caudalmiddlefrontal | 1.38(0.17) | 0.03(0.97) | -1.20(0.23) | 0.44(0.66) | -0.47(0.64) | -1.14(0.16) |
| L_cuneus | 1.10(0.27) | 1.81(0.07) | -0.66(0.51) | -0.85(0.40) | 0.21(0.84) | 0.70(0.49) |
| L_entorhinal | 1.23(0.22) | -0.38(0.71) | -1.20(0.23) | -0.95(0.35) | 1.65(0.11) | -1.51(0.14) |
| L_fusiform | 1.11(0.27) | -0.55(0.58) | 0.07(0.94) | 0.64(0.53) | 0.25(0.80) | -0.11(0.91) |
| L_inferiorparietal | -1.19(0.24) | 1.94(0.05) | -0.94(0.35) | 0.96(0.34) | -0.68(0.50) | 0.32(0.75) |
| L_inferiortemporal | 1.08(0.28) | -0.28(0.78) | -0.28(0.78) | 0.22(0.82) | -0.43(0.67) | 0.56(0.58) |
| L_isthmuscingulate | -0.25(0.80) | 0.31(0.76) | -1.04(0.30) | 0.40(0.69) | -0.47(0.64) | -0.48(0.63) |
| L_lateraloccipital | -1.33(0.19) | 0.96(0.34) | 0.64(0.53) | -0.11(0.91) | -1.31(0.20) | 0.74(0.46) |
| L_lateralorbitofrontal | **3.71(2.9****×10^-4^)** | -0.89(0.38) | -0.62(0.54) | 1.37(0.18) | -0.78(0.44) | -0.69(0.49) |
| L_lingual | 1.40(0.17) | 0.57(0.57) | -1.15(0.25) | 0.95(0.35) | -0.36(0.72) | 0.06(0.96) |
| L_medialorbitofrontal | 1.60(0.11) | -1.09(0.28) | -0.39(0.70) | -0.48(0.64) | -0.30(0.77) | -1.98(0.05) |
| L_middletemporal | 0.48(0.63) | 0.76(0.45) | -0.92(0.36) | 1.23(0.22) | -0.69(0.49) | -0.10(0.92) |
| L_parahippocampal | -1.36(0.18) | 1.05(0.30) | 0.11(0.91) | 1.08(0.28) | 0.23(0.82) | -0.55(0.59) |
| L_paracentral | -0.62(0.54) | -0.06(0.95) | -1.03(0.30) | 0.92(0.36) | -0.86(0.40) | -0.82(0.42) |
| L_parsopercularis | 0.92(0.36) | -0.73(0.47) | 0.36(0.72) | 0.20(0.84) | -1.01(0.32) | 0.07(0.95) |
| L_parsorbitalis | 1.65(0.10) | 0.13(0.90) | -0.56(0.58) | -0.67(0.51) | -0.02(0.99) | 0.09(0.93) |
| L_parstriangularis | 1.35(0.18) | 0.09(0.93) | 0.21(0.84) | -0.58(0.57) | 1.55(0.13) | -1.30(0.20) |
| L_pericalcarine | -0.58(0.57) | 0.59(0.55) | -0.84(0.40) | 1.08(0.28) | 0.94(0.35) | -0.15(0.88) |
| L_postcentral | -1.61(0.11) | -0.89(0.37) | 0.74(0.46) | -0.24(0.82) | -0.35(0.73) | 0.59(0.56) |
| L_posteriorcingulate | -0.003(1.0) | -1.99(0.05) | 0.53(0.60) | 0.77(0.44) | -1.14(0.26) | -0.22(0.83) |
| L_precentral | 0.50(0.62) | 0.22(0.82) | -1.75(0.08) | 1.51(0.14) | -0.01(0.99) | -1.87(0.07) |
| L_precuneus | -0.11(0.91) | 0.30(0.76) | -0.17(0.87) | 0.49(0.63) | -0.89(0.38) | 0.22(0.83) |
| L_rostralanteriorcingulate | 1.51(0.13) | -0.93(0.35) | -0.93(0.35) | -1.73(0.09) | 0.28(0.78) | -0.67(0.51) |
| L_rostralmiddlefrontal | 1.24(0.22) | 0.68(0.50) | -0.32(0.75) | -0.20(0.84) | -0.25(0.80) | 0.08(0.94) |
| L_superiorfrontal | 1.28(0.20) | 0.87(0.38) | -0.49(0.62) | 1.28(0.21) | -0.74(0.46) | -1.30(0.20) |
| L_superiorparietal | -1.33(0.18) | 0.95(0.34) | -0.66(0.51) | -0.02(0.99) | -1.47(0.15) | 0.73(0.47) |
| L_superiortemporal | 0.58(0.56) | 0.54(0.59) | -1.52(0.13) | 0.25(0.80) | -0.60(0.55) | 0.42(0.68) |
| L_supramarginal | -0.82(0.42) | -0.42(0.67) | -0.26(0.80) | 0.49(0.63) | -0.53(0.60) | 0.26(0.80) |
| L_frontalpole | **2.30(0.02)** | 1.75(0.08) | **-2.21(0.03)** | 1.01(0.32) | -1.03(0.31) | -1.76(0.08) |
| L_temporalpole | 0.22(0.82) | 0.45(0.65) | -1.36(0.18) | 0.72(0.47) | -0.79(0.44) | -0.26(0.80) |
| L_transversetemporal | -0.04(0.97) | -0.48(0.63) | -1.29(0.20) | 0.74(0.47) | 0.45(0.66) | -1.67(0.10) |
| L_insula | **2.75(0.007)** | -0.09(0.93) | -1.65(0.10) | 0.32(0.75) | 0.25(0.81) | -1.48(0.15) |
| R_bankssts | -0.36(0.72) | -1.93(0.06) | -0.02(0.98) | 1.01(0.32) | -1.14(0.26) | 1.07(0.29) |
| R_caudalanteriorcingulate | 0.23(0.81) | 1.31(0.19) | 1.47(0.15) | 1.84(0.07) | 0.13(0.90) | -0.52(0.61) |
| R_caudalmiddlefrontal | **2.16(0.03)** | 1.05(0.30) | -1.62(0.11) | 0.62(0.54) | 0.46(0.65) | -0.96(0.34) |
| R_cuneus | 1.21(0.22) | 0.77(0.44) | -0.37(0.71) | -1.57(0.12) | -1.15(0.26) | 1.37(0.18) |
| R_entorhinal | 1.79(0.08) | 0.72(0.47) | -1.06(0.29) | -0.51(0.61) | -0.04(0.97) | -0.15(0.88) |
| R_fusiform | 1.15(0.25) | 0.03(0.98) | -1.15(0.25) | -0.03(0.98) | 0.61(0.54) | -0.57(0.57) |
| R_inferiorparietal | -1.78(0.08) | 0.37(0.71) | 0.31(0.76) | 1.46(0.15) | -0.49(0.63) | 0.67(0.50) |
| R_inferiortemporal | 1.86(0.07) | -0.24(0.81) | 0.03(0.98) | 0.62(0.54) | -0.99(0.32) | 1.68(0.10) |
| R_isthmuscingulate | -0.04(0.97) | 1.32(0.19) | -1.01(0.32) | 0.11(0.91) | 1.15(0.26) | -0.86(0.39) |
| R_lateraloccipital | **-2.01(0.05)** | 0.84(0.40) | 0.52(0.60) | 0.76(0.45) | -0.60(0.55) | 0.45(0.65) |
| R_lateralorbitofrontal | **2.89(0.004)** | -0.14(0.89) | -1.18(0.24) | 0.92(0.36) | 0.96(0.34) | **-2.20(0.03)** |
| R_lingual | **2.35(0.02)** | 0.36(0.72) | -0.82(0.41) | -0.003(1.0) | -0.16(0.88) | 0.52(0.61) |
| R_medialorbitofrontal | 1.78(0.08) | 0.38(0.70) | -1.46(0.15) | 0.77(0.45) | 0.65(0.52) | -0.12(0.91) |
| R_middletemporal | 0.83(0.41) | 0.18(0.86) | -0.62(0.53) | 1.15(0.26) | -0.95(0.35) | 0.05(0.96) |
| R_parahippocampal | -0.13(0.90) | 0.41(0.68) | -0.14(0.89) | 0.93(0.36) | 0.09(0.93) | 0.13(0.90) |
| R_paracentral | 0.49(0.62) | 0.95(0.34) | -1.85(0.07) | 0.59(0.56) | 0.04(0.97) | -0.74(0.46) |
| R_parsopercularis | 0.36(0.72) | 0.73(0.47) | -0.43(0.67) | 1.89(0.06) | 0.69(0.49) | -1.12(0.27) |
| R_parsorbitalis | 1.67(0.10) | 0.62(0.54) | -0.004(1.0) | 1.73(0.09) | 0.23(0.82) | -0.23(0.82) |
| R_parstriangularis | 0.76(0.45) | 0.36(0.74) | -0.69(0.50) | -0.13(0.90) | 0.19(0.85) | 0.05(0.96) |
| R_pericalcarine | 0.76(0.45) | 0.71(0.48) | -1.84(0.07) | -0.83(0.41) | 1.36(0.18) | -2.60(0.01) |
| R_postcentral | -1.53(0.13) | 0.03(0.97) | -0.05(0.96) | 1.40(0.17) | -1.63(0.11) | 1.04(0.30) |
| R_posteriorcingulate | -0.57(0.57) | 0.64(0.52) | -0.39(0.70) | 0.79(0.43) | 0.07(0.95) | -1.33(0.19) |
| R_precentral | 0.50(0.62) | -0.33(0.75) | -1.06(0.29) | 0.30(0.76) | 0.08(0.94) | -1.12(0.27) |
| R_precuneus | 0.54(0.59) | 0.54(0.59) | -0.30(0.76) | -0.27(0.79) | -1.39(0.17) | 1.49(0.14) |
| R_rostralanteriorcingulate | 1.92(0.06) | -0.73(0.47) | 0.32(0.75) | 1.39(0.17) | 0.04(0.97) | -1.56(0.13) |
| R_rostralmiddlefrontal | 0.79(0.43) | 1.03(0.31) | -1.25(0.21) | 0.39(0.70) | -0.71(0.48) | -0.44(0.66) |
| R_superiorfrontal | 1.13(0.26) | 1.43(0.16) | -0.79(0.43) | 0.07(0.94) | 0.30(0.77) | -1.04(0.30) |
| R_superiorparietal | -1.45(0.15) | 0.61(0.54) | -0.09(0.93) | -0.29(0.77) | -1.63(0.11) | 1.83(0.07) |
| R_superiortemporal | 1.76(0.08) | 0.24(0.81) | -1.40(0.17) | 0.85(0.40) | -0.68(0.50) | -0.32(0.75) |
| R_supramarginal | -1.27(0.21) | -0.72(0.47) | 0.38(0.71) | 0.05(0.96) | -0.56(0.58) | 0.46(0.65) |
| R_frontalpole | **2.24(0.03)** | 1.27(0.21) | -0.79(0.43) | 2.18(0.03) | -0.006(1.0) | -0.99(0.33) |
| R_temporalpole | 1.24(0.22) | 1.06(0.29) | -1.42(0.16) | 0.17(0.86) | -0.46(0.65) | 0.02(0.99) |
| R_transversetemporal | 0.41(0.68) | -1.07(0.28) | **-2.48(0.01)** | -1.14(0.26) | **-2.27(0.03)** | -0.18(0.86) |
| R_insula | 0.85(0.40) | 0.39(0.70) | -1.70(0.29) | 0.37(0.71) | 0.44(0.66) | -1.10(0.27) |

Variance reported as *t* value (*p* value). Abbreviations: 5-HI: 5-hydroxyindole; KYNA: Kynurenic acid; QUIN: Quinolinic acid; L: left; R: right.

**Supplementary table 5. Multiple regression analysis results of left** **lateral orbitofrontal cortex on working memory and other MCCB scores adjusting for sex and age**

|  |  | Model | Left LOFC | Sex | Age |
| --- | --- | --- | --- | --- | --- |
|  |  | *F* Value | *t* Value | *t* Value | *t* Value |
| Group | Domain | *p* Value | *p* Value | *p* Value | *P* Value |
| Schizophrenia | Working memory | **4.17** | **2.68** | 2.62 | 0.01 |
|  |  | **0.007** | **0.008** | 0.01 | 0.18 |
|  | Processing speed | 0.45 | 1.02 | 0.65 | 0.46 |
|  |  | 0.72 | 0.31 | 0.52 | 0.65 |
|  | Attention/vigilance | 2.15 | 0.80 | 0.28 | 2.40 |
|  |  | 0.10 | 0.43 | 0.78 | 0.02 |
|  | Verbal learning | **6.88** | **4.17** | 2.13 | 3.36 |
|  |  | **<0.001** | **<0.001** | 0.04 | 0.001 |
|  | Visual learning | 4.82 | 1.49 | 3.57 | 0.38 |
|  |  | 0.003 | 0.14 | <0.001 | 0.71 |
|  | Reasoning and problem solving | **11.81** | **2.47** | 1.10 | -2.72 |
|  |  | **<0.001** | **0.02** | 0.29 | 0.007 |
|  | Social cognition | **4.44** | **3.05** | 1.03 | 0.38 |
|  |  | **0.005** | **0.003** | 0.31 | 0.71 |
| Healthy controls | Working memory | 0.78 | 0.11 | 1.53 | 0.01 |
|  |  | 0.51 | 0.92 | 0.13 | 0.99 |
|  | Processing speed | 0.09 | 0.25 | 0.01 | 0.50 |
|  |  | 0.97 | 0.80 | 0.99 | 0.62 |
|  | Attention/vigilance | 0.31 | -0.23 | 0.59 | 0.62 |
|  |  | 0.82 | 0.82 | 0.56 | 0.54 |
|  | Verbal learning | 0.33 | -2.23 | 0.33 | 0.81 |
|  |  | 0.81 | 0.82 | 0.74 | 0.42 |
|  | Visual learning | 1.28 | 0.73 | 0.19 | -1.59 |
|  |  | 0.29 | 0.47 | 0.85 | 0.12 |
|  | Reasoning and problem solving | 1.24 | 1.52 | 0.82 | -0.63 |
|  |  | 0.30 | 0.13 | 0.41 | 0.53 |
|  | Social cognition | 1.03 | 1.20 | -1.86 | -0.35 |
|  |  | 0.41 | 0.23 | 0.07 | 0.73 |

**Supplementary material 6. The relationship between subcortical regions volume with serum levels of KYNA and QUIN/KYNA.**

**6.1** **MRI protocol**

Following the ENIGMA protocol (<http://enigma.ini.usc.edu/>), Hippocampus, Amygdala, Pallidum, Putamen, Caudate, Lateral ventricle, Thalamus volume were obtained with FreeSurfer ^1, 2^ (http://surfer.nmr.mgh.harvard.edu).

**6.2 Results**

Multiple subcortical segmentations showed significantly reduced cortical thickness in patients compared to controls (**Supplementary table 6.1**). We further investigate the relationship between subcortical segmentations and KYNA levels, and KYNA/QUIN respectively. However, there was no significant results in patients or healthy controls (all p’s>0.05) (**Supplementary table 6.2**).

**Supplementary table 6.1 A comparison of subcortical volume between schizophrenia patients and healthy controls by ANOVA**

|  | Healthy controls | | Schizophrenia | | *F* value | *P* value |
| --- | --- | --- | --- | --- | --- | --- |
| Subcortical regions | Mean（m^3^） | SEM | Mean（m^3^） | SEM |  |  |
| Left Hippocampus | 3966.7 | 40.4 | 3880.4 | 31.7 | 2.33 | 0.13 |
| Right Hippocampus | 4176.6 | 41.0 | 4044.0 | 34.4 | 4.78 | 0.03 |
| Left Amygdala | 1670.0 | 21.8 | 1560.0 | 24.8 | 15.28 | <0.001 |
| Right Amygdala | 1741.2 | 24.8 | 1677.5 | 15.3 | 4.84 | 0.03 |
| Left Pallidum | 2065.5 | 26.3 | 2145.3 | 23.3 | 3.86 | 0.05 |
| Right Pallidum | 2027.6 | 27.6 | 2102.7 | 21.1 | 3.93 | 0.05 |
| Left Putamen | 4981.3 | 72.7 | 5061.2 | 58.1 | 0.60 | 0.44 |
| Right Putamen | 5081.8 | 72.4 | 5097.3 | 54.7 | 0.03 | 0.88 |
| Left Caudate | 3383.8 | 53.6 | 3568.6 | 38.9 | 6.85 | 0.01 |
| Right Caudate | 3425.7 | 53.1 | 3599.8 | 41.9 | 5.43 | 0.02 |
| Left Lateral Ventricle | 7782.6 | 605.5 | 9874.4 | 398.9 | 8.76 | 0.003 |
| Right Lateral Ventricle | 6604.3 | 410.1 | 8461.4 | 354.0 | 8.96 | 0.003 |
| Left Thalamus Proper | 7883.3 | 110.5 | 8656.4 | 140.5 | 10.87 | 0.001 |
| Right Thalamus Proper | 7409.9 | 105.5 | 8095.3 | 119.3 | 11.58 | 0.001 |

**Supplementary table 6.2** **The relationship between subcortical regions volume with serum levels of KYNA and QUIN/KYNA controlling for age and sex.**

|  | Healthy controls | | Schizophrenia | |
| --- | --- | --- | --- | --- |
| Subcortical regions | KYNA  *r* value (*p* value) | QUIN/KYNA  *r* value (*p* value) | KYNA  *r* value (*p* value) | QUIN/KYNA  *r* value (*p* value) |
| Left Hippocampus | -0.21 (0.09) | 0.22 (0.08) | -0.14 (0.86) | 0.01 (0.96) |
| Right Hippocampus | -0.12 (0.36) | 0.12 (0.35) | -0.01 (0.94) | 0.08 (0.31) |
| Left Amygdala | -0.09 (0.48) | 0.12 (0.34) | -0.05 (0.56) | -0.03 (0.72) |
| Right Amygdala | -0.02 (0.89) | 0.07 (0.57) | -0.05 (0.53) | 0.12 (0.14) |
| Left Pallidum | -0.01(0.96) | 0.18 (0.17) | 0.02 (0.76) | -0.02 (0.80) |
| Right Pallidum | -0.02 (0.89) | 0.19 (0.15) | 0.08 (0.33) | -0.06 (0.45) |
| Left Putamen | 0.09 (0.48) | 0.07 (0.58) | 0.06 (0.42) | 0.04 (0.60) |
| Right Putamen | 0.06 (0.61) | 0.06 (0.64) | 0.05 (0.51) | 0.04 (0.61) |
| Left Caudate | 0.07 (0.60) | 0.08 (0.56) | 0.11 (0.15) | -0.06 (0.48) |
| Right Caudate | 0.03 (0.85) | 0.09 (0.51) | 0.08 (0.30) | -0.05 (0.52) |
| Left Lateral Ventricle | -0.02 (0.90) | -0.05 (0.68) | 0.05 (0.56) | -0.11 (0.18) |
| Right Lateral Ventricle | 0.01 (0.96) | -0.05 (0.71) | 0.06 (0.43) | -0.13 (0.09) |
| Left Thalamus Proper | -0.14 (0.29) | 0.10 (0.44) | -0.11 (0.16) | 0.10 (0.22) |
| Right Thalamus Proper | -0.16 (0.22) | 0.11 (0.40) | -0.12 (0.14) | 0.10 (0.19) |

**Supplementary material 7. Differentiation of 5-hydroxyindole (5-HI) and 5-hydroxyoxindole (5-HOI)**

Some studies reported that 5-HI and 5-HOI were two different metabolites in the pathway of tryptophan metabolisms (Figure 1), and had different physiological properties ^3-5^. However, one study mentioned that 5-HI was not produces via a known metabolic pathway but is produced by a synthetic modification of indole ^6^. To the best of our knowledge, 5-HI and 5-HOI might not have been examined together in human blood samples. Thus, we tested the 5-HOI 5-HI to provide evidences they both could be tested in the serum, and that the reported findings are associated with serum 5-HI but not 5-HOI.

It became clear to us that detection of serum 5-HOI using the same pretreatment method as 5-HI is challenging because of the low levels. The proper multiple reaction monitoring (MRM) transition of 5-HOI is specified as 150.02/77.18, which is distinct from the transition 134.16/106.41 for 5-HI analysis. In order to detect the low level of 5-HOI in serum, a higher sensitivity method is needed.

We chose trichloroacetic acid (TCA) was used for protein precipitation, which can provide better accumulation of 5-HOI from the serum compared to the organic solvent. Note that 5-HI and 5-HOI have different molecular weight and they go into different ion channels due to their different parent ions and daughter ions (showed in supplementary figure 7.1). These properties can also help to distinguish them. We tested 5-HOI in some participants with serum sample available (*n*=28). Methods are described below. Results showed that 5-HOI levels were insignificantly different between patients with schizophrenia (mean [SEM], 2.39 [0.61] ng/mL) and HC (mean [SEM], 3.53 [0.67] ng/mL) with Mann-Whitney test (*Z* = 1.38, *p* = 0.17). No significant associations between 5-HOI and 5-HI were found in patients (*p* = 0.38), controls (*p* = 0.44), or in total subjects (*p* = 0.59). Therefore, we were confident that the data reported in this manuscript is 5-HI and not 5-HOI. More details of the 5-HOI assaying methods are described below. (Differences pretreatment from 5-HI were highlighted in blue):

**7.1 Materials and Methods**

**7.1.1 Chemicals and reagents**

5-hydroxyoxindole (5-HOI, purity 99%) was obtained from the Sigma reagent company (Darmstadt, Germany). 5-hydroxytryptamine-d4 HCl (5-HT-d4, purity >98%) was purchased from ISOREAG standards (Shanghai, China). Formic acid and methanol were acquired from Merck (Darmstadt, Germany). Purified water was obtained from A.S. Watson Group (Hongkong, China).

**7.1.2 Chromatographic and mass spectrometric conditions**

A Waters Acquity UPLC I-class system (Waters, Shanghai) equipped with Waters Xevo TQS IVD system (Waters, Shanghai) with ESI source was used. The chromatographic separation was achieved on a Waters Acquity UPLC HSS T3 (2.1 × 50 mm, 1.8 μm) column with a column temperature of 35℃. A gradient elution with a flow rate of 0.3 mL/min using 0.1% Formic Acid (FA) and 2mM Ammonium Formate in water as solvent A and 0.1% FA in acetonitrile as solvent B was performed. The elution program was set as follows: 0-0.7 min 5% B, 0.7-1 min 5%-20% B, 1-1.65 min 20% B, 1.65-1.7 min 20%-5% B, 1.7-2 min 5% B. The sampler chamber temperature kept at 6℃.

**7.1.3 Mass Spectrometric parameters**

The mass detection and quantification were performed in a positive ion mode. The mass spectrometer working parameters were optimized as follows: capillary voltage of 3.5 kV, ion source temperature of 150℃, desolvation temperature of 550℃, desolvation gas flow of 800 L/Hr and cone gas flow of 150 L/Hr. Multipole reaction monitoring (MRM) mode was used for the analysis with mass transition parameters as follows:

**Supplementary table 7.1. Mass transition parameters**

| **Analytes** | **parent(m/z)** | **daughter(m/z)** | **Dwell(s)** | **cone(V)** | **collision(V)** |
| --- | --- | --- | --- | --- | --- |
| **5-HOI**  **(Qualitative analysis)** | 150.02 | 77.18 | 0.079 | 20 | 25 |
| **5-HOI**  **（Quantitative analysis）** | 150.06 | 95.03 | 0.079 | 20 | 25 |
| **5-HT-d4** | 181.16 | 163.96 | 0.079 | 10 | 8 |

**7.1.4 Calibrators and quality controls**

An aliquot of 10 µL different working solutions was spiked with 90 µL 50% methanol/water aliquot separately and vortexed for 1 min to prepare calibration standard samples at different concentration levels. The calibrator concentrations were 1, 2.5, 10, 50, 100, 500 and 1000 ng/mL for 5-HOI. The QC samples were prepared as the same procedures to give different concentrations of 5-HOI at 2.5 and 10 ng/mL.

**7.1.5 Preparation of samples**

An aliquot of 10 μL internal standard (IS) solution (800 ng/mL 5-HT-d4 in methanol) and 150 μL TCA were added to 100 μL sample and vortexed for 3 min. The mixture was then centrifuged at 4℃ and 15000 rpm for 10 min. 100 μL supernatant was then transferred to a clean vial with the addition of 100 μL pure water and vortexed for 1 min. The mixture was then followed by centrifugation at 4℃ and 15000 rpm for 5 min. The 100 μL supernatant was finally injected into the LC-MS/MS system.

**7.1.6 Validation of the methods**

The established method was validated for specificity, linearity, accuracy and precision.

7.1.6.1 Selectivity.

Selectivity of the method was assessed by observing the interference at the retention time of analysts and IS in plasma sample.

7.1.6.2 Calibration curve linearity.

The calibration curve was constructed by plotting the peak area ratios of each analyte/IS versus its nominal concentrations, using a linear regression.

7.1.6.3 Accuracy and precision.

The replicates (n=5) of QC samples were analyzed to determine the precision and accuracy of the method. The precision is expressed by CV between the replicate measurements. Accuracy is defined as relative error (RE) which is calculated using the formula RE% = [(measured value−theoretical value)/ theoretical value] ×100%.

**7.2 Results**

**7.2.1 Selectivity**

The MRM chromatograms of analytes were presented in Figure 7.1. The peak shapes of all the analytes were of good quality for assay. The total ion chromatography of plasma sample, the blank 50% methanol solution and QC sample were shown in Figure 7.1. No significant chromatographic peak area interference was observed either at the retention time of analytes or IS in blank 50% methanol solution.

**Supplementary figure 7.1. The MRM chromatograms of 5-HOI in the blank 50% methanol solution (A), QC sample (B) and serum sample(C).**


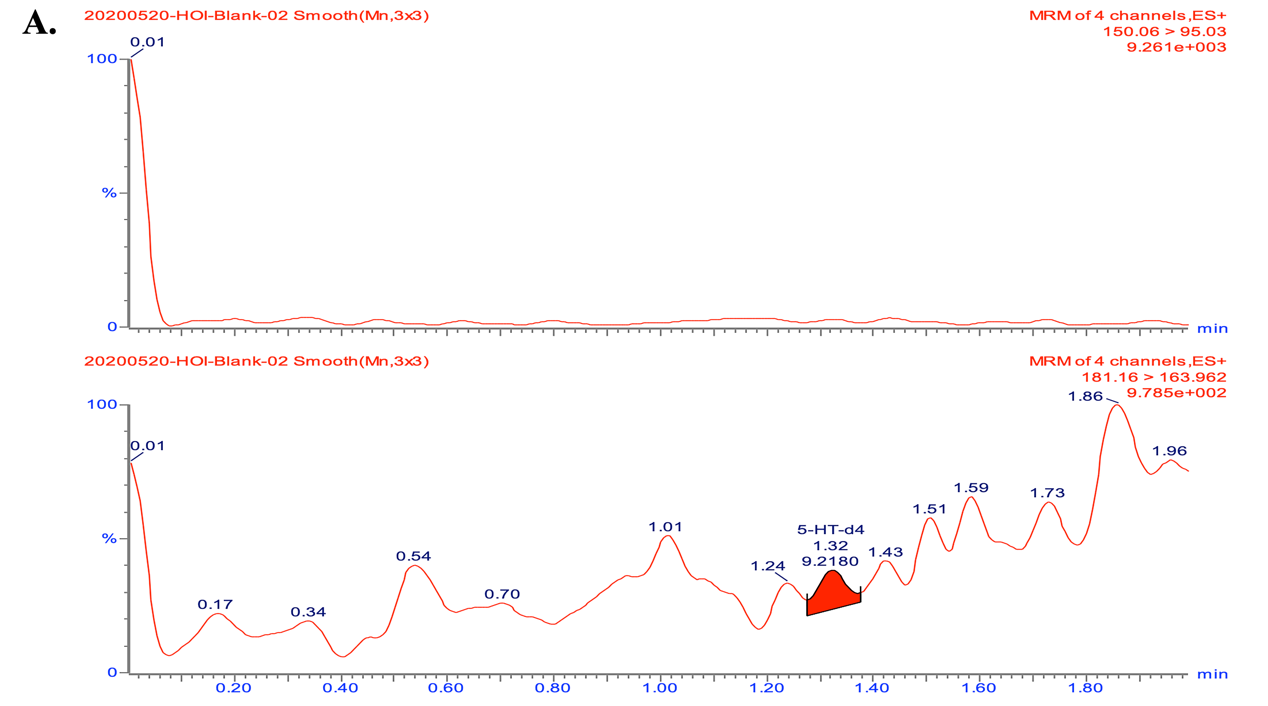

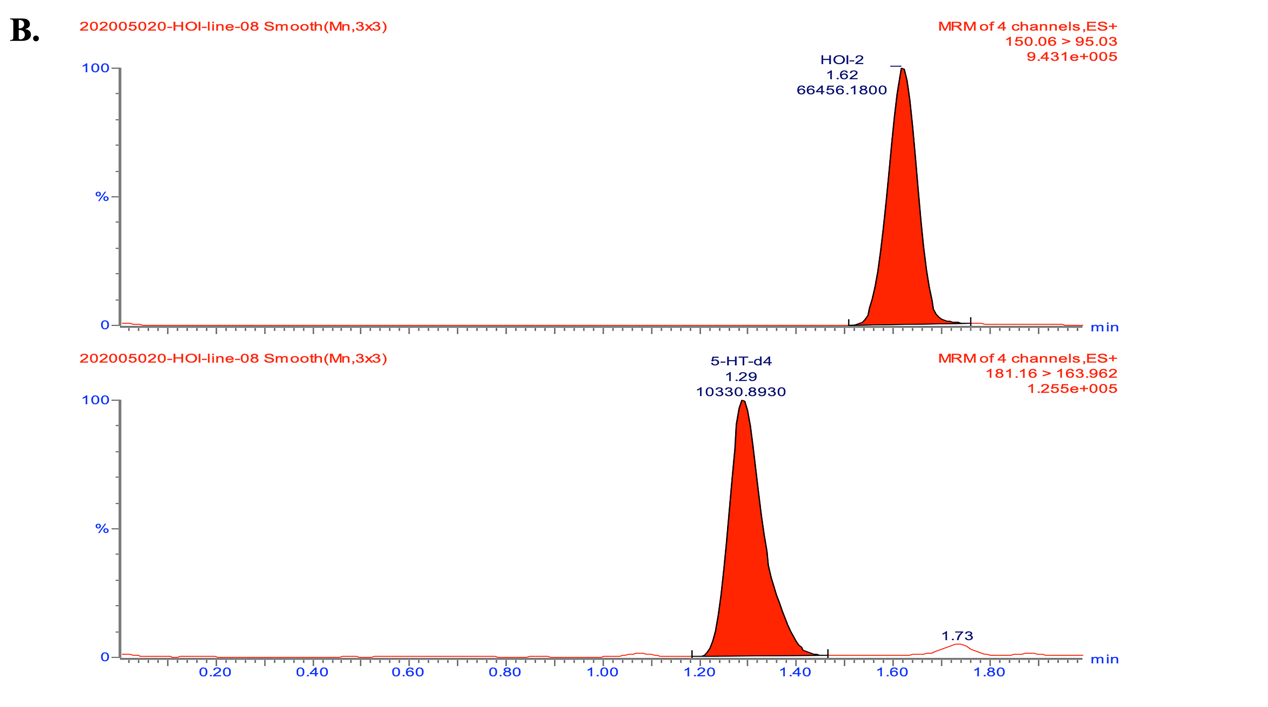


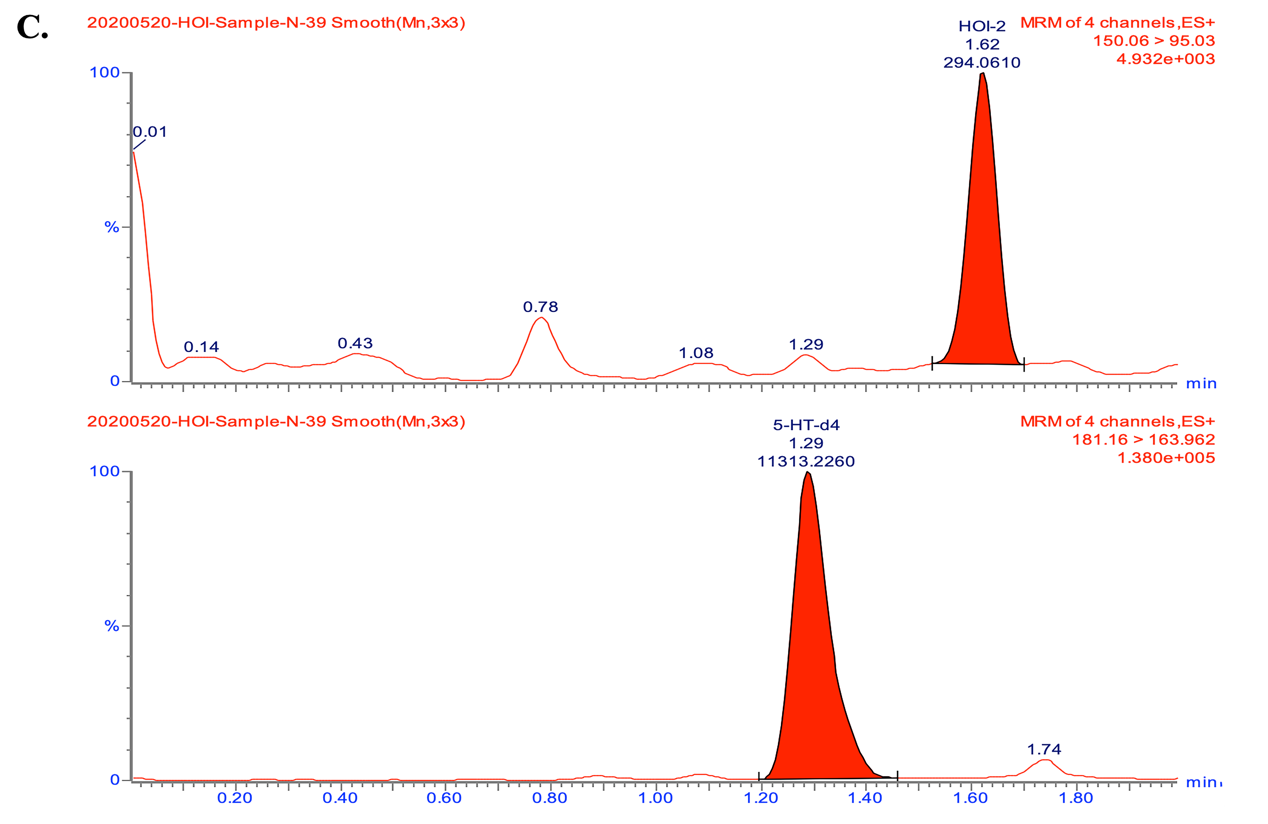


**7.2.2 Calibration curve linearity.**

The calibration curves are showed in Figure 7.2. all calibration curves exhibited good linearity with correlation coefficient (r^2^) within the range of 0.9902–0.9998.

**Supplementary figure 7.2 The calibration curve for 5-HOI.**

**
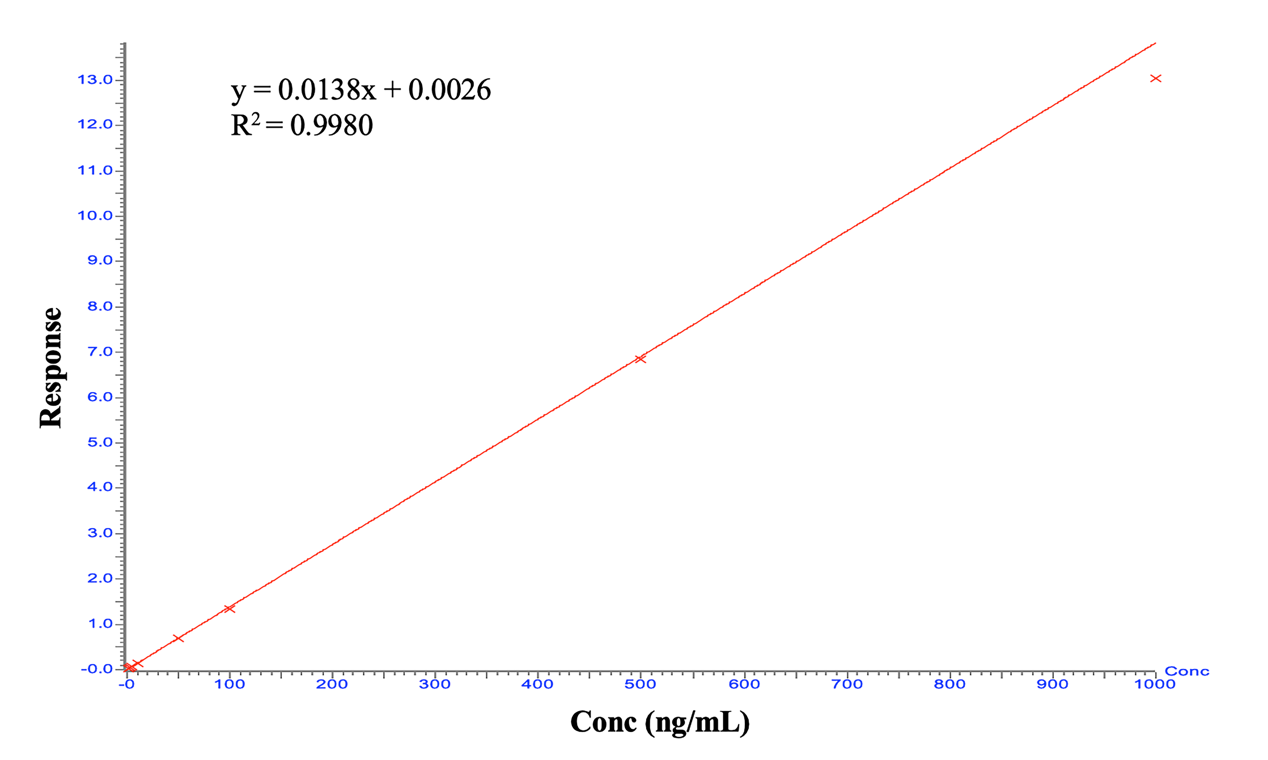
**

**7.2.3 Accuracy and precision.**

As presented in the Table 7.2, the precision (CV) ranged between -3.5 and 11.2 %, and accuracy (RE) ranged between -5.6 and -13.6 %, which were acceptable for biological analysis.

**Supplementary table 7.2. The precision and accuracy of the method.**

| **Analytes** | **Nominal Conc (ng/mL)** | **CV（%）** | **RE（%）** |
| --- | --- | --- | --- |
| **5-HOI** | 2.5 | 11.2 | -13.6 |
|  | 10 | -3.5 | -5.6 |

**References**

1. Fischl B. FreeSurfer. *NeuroImage* 2012; **62**(2)**:** 774-781.

2. Fischl B *et al.* Whole brain segmentation: automated labeling of neuroanatomical structures in the human brain. *Neuron* 2002; **33**(3)**:** 341-355.

3. Mannaioni G, Carpenedo R, Moroni F. 5-hydroxyindole causes convulsions and increases transmitter release in the CA1 region of the rat hippocampus. *British journal of pharmacology* 2003; **138**(1)**:** 245-253.

4. King LJ, Parke DV, Williams RT. The metabolism of [2-14C] indole in the rat. *The Biochemical journal* 1966; **98**(1)**:** 266-277.

5. Moroni F *et al.* Studies on the pharmacological properties of oxindole (2-hydroxyindole) and 5-hydroxyindole: are they involved in hepatic encephalopathy? *Advances in experimental medicine and biology* 1997; **420:** 57-73.

6. Papy-Garcia D *et al.* Detection and quantification of 5-hydroxyoxindole in mammalian sera and tissues by high performance liquid chromatography with multi-electrode electrochemical detection. *Clinical biochemistry* 2003; **36**(3)**:** 215-220.
